# Timely administration of tocilizumab improves outcome of hospitalized COVID-19 patients

**DOI:** 10.1371/journal.pone.0271807

**Published:** 2022-08-12

**Authors:** Abraham Rutgers, Peter E. Westerweel, Bronno van der Holt, Simone Postma, Marit G. A. van Vonderen, Djura P. Piersma, Douwe Postma, Maarten van den Berge, Eefje Jong, Marten de Vries, Leonie van der Burg, Dennis Huugen, Marjolein van der Poel, Linda M. Kampschreur, Marcel Nijland, Jaap H. Strijbos, Menno Tamminga, Pim G. N. J. Mutsaers, Suzanne Schol-Gelok, Margriet Dijkstra-Tiekstra, Grigory Sidorenkov, Julien Vincenten, Wouter H. van Geffen, Marjolein Knoester, Jos Kosterink, Reinold Gans, Coen Stegeman, Gerwin Huls, Tom van Meerten

**Affiliations:** 1 Department of Rheumatology and Clinical Immunology, University Medical Center Groningen, Groningen, The Netherlands; 2 Department of Internal Medicine, Albert Schweitzer Hospital, Dordrecht, The Netherlands; 3 HOVON Data Center, Department of Haematology, Erasmus MC Cancer Institute, Rotterdam, The Netherlands; 4 Department of Haematology, University Medical Center Groningen, Groningen, The Netherlands; 5 Department of Internal Medicine, Medical Center Leeuwarden, Leeuwarden, The Netherlands; 6 Department of Internal Medicine, Medisch Spectrum Twente, Enschede, The Netherlands; 7 Department of Internal Medicine, University Medical Center Groningen, Groningen, The Netherlands; 8 Department of Pulmonology, University Medical Center Groningen, Groningen, The Netherlands; 9 Department of Infectious Diseases, Meander MC, Amersfoort, The Netherlands; 10 Department of Pulmonology, Tjongerschans Hospital, Heerenveen, The Netherlands; 11 Department of Internal Medicine, Antonius Hospital Sneek, Sneek, The Netherlands; 12 Department of Internal Medicine, Deventer Hospital, Deventer, The Netherlands; 13 Department of Internal Medicine, Maastricht University Medical Centre+, Maastricht, The Netherlands; 14 Department of Pulmonology, Nij Smellinghe Hospital, Drachten, The Netherlands; 15 Department of Haematology, Erasmus Medical Center, Rotterdam, The Netherlands; 16 Department of Emergency Medicine, Albert Schweitzer Hospital, Dordrecht, The Netherlands; 17 Department of Epidemiology, University Medical Center Groningen, Groningen, The Netherlands; 18 Department of Pulmonology, Albert Schweitzer Hospital, Dordrecht, The Netherlands; 19 Department of Pulmonology, Medical Center Leeuwarden, Leeuwarden, The Netherlands; 20 Department of Medical Microbiology, University Medical Center Groningen, Groningen, The Netherlands; 21 Pharmacy, University Medical Center Groningen, Groningen, The Netherlands; Amsterdam UMC - Locatie VUMC: Amsterdam UMC Locatie VUmc, NETHERLANDS

## Abstract

**Introduction:**

The aim of this study was to determine the efficacy of early tocilizumab treatment for hospitalized patients with COVID-19 disease.

**Methods:**

Open-label randomized phase II clinical trial investigating tocilizumab in patients with proven COVID-19 admitted to the general ward and in need of supplemental oxygen. The primary endpoint of the study was 30-day mortality with a prespecified 2-sided significance level of α = 0.10. A post-hoc analysis was performed for a combined endpoint of mechanical ventilation or death at 30 days. Secondary objectives included comparing the duration of hospital stay, ICU admittance and duration of ICU stay and the duration of mechanical ventilation.

**Results:**

A total of 354 patients (67% men; median age 66 years) were enrolled of whom 88% received dexamethasone. Thirty-day mortality was 19% (95% CI 14%-26%) in the standard arm versus 12% (95% CI: 8%-18%) in the tocilizumab arm, hazard ratio (HR) = 0.62 (90% CI 0.39–0.98; p = 0.086). 17% of patients were admitted to the ICU in each arm (p = 0.89). The median stay in the ICU was 14 days (IQR 9–28) in the standard arm versus 9 days (IQR 5–14) in the tocilizumab arm (p = 0.014). Mechanical ventilation or death at thirty days was 31% (95% CI 24%-38%) in the standard arm versus 21% (95% CI 16%-28%) in the tocilizumab arm, HR = 0.65 (95% CI 0.42–0.98; p = 0.042).

**Conclusions:**

This randomized phase II study supports efficacy for tocilizumab when given early in the disease course in hospitalized patients who need oxygen support, especially when concomitantly treated with dexamethasone.

**Trial registration:**

https://www.trialregister.nl/trial/8504.

## Introduction

Severe acute respiratory syndrome coronavirus 2 (SARS-CoV-2) causes coronavirus disease 2019 (Covid-19). While many people infected with Covid-19 will experience only mild or uncomplicated symptoms, up to 15% of patients develop more serious symptoms that require hospitalization with oxygen support, and up to 5% of patients will need mechanical ventilation or organ support in an intensive care unit (ICU) [1–4]. Among Covid-19 patients requiring mechanical ventilation, the duration of ventilation is significant and mortality high. It is therefore imperative to understand and therapeutically target pathological factors involved in respiratory failure. In most patients, serious symptoms develop late in the disease course, when the SARS-CoV-2 viral load is decreasing. Dysregulation of the host immune response upon SARS-CoV-2 infection may lead to sepsis-like symptoms and respiratory distress with hypoxemia. Indeed, pro-inflammatory cytokines such as interleukin-6 (IL-6), Il-1β and inflammatory parameters such as C-reactive protein (CRP) and ferritin are often elevated in patients with Covid-19 [2, 5, 6]. In addition, administration of glucocorticoids, which curtail the host immune response, reduces Covid-19-related mortality [7]. The multisystem inflammatory syndrome in patients with serious Covid-19 resembles cytokine release syndrome (CRS) [8, 9], which has been observed in cancer patients treated with immunotherapies such as chimeric antigen receptor (CAR) T-cell therapy [10].

As high IL-6 levels are strongly associated with shorter survival in Covid-19 [6] and IL-6 signalling can be efficiently blocked by the IL-6 inhibitor tocilizumab, we hypothesized that early (pre-emptive) intervention with tocilizumab might beneficially alter the course of Covid-19. We postulated that tocilizumab would reduce progression to hypoxemic respiratory failure and death, reduce the risk of admission to the intensive care unit (ICU) and decrease the duration of ICU and hospital stay. To this end we designed and carried out an investigator-initiated trial, the ‘Pre-emptive Tocilizumab for hospitalized Covid-19 patients’ (PreToVid) trial. This prospective randomized trial compared standard of care with or without tocilizumab in Covid-19 patients admitted to hospital and in need of oxygen supplementation.

## Methods

### Trial design

The trial was designed as a prospective randomized (1:1) open label phase II trial and was registered in the Netherlands Trial register (https://www.trialregister.nl/trial/8504). Eleven Dutch hospitals participated, consisting of 8 regional hospitals and 3 academic centres. The trial was approved by Medical Ethical Committee of the University Medical Centre Groningen (reference METc 2020/172) and was performed in accordance with Good Clinical Practice guidelines and the Helsinki Declaration. All patients provided written informed consent. The investigators designed the trial, collected the data and performed all analyses. Although tocilizumab was provided free of charge by Roche, the company had no role in the design of the study, data analysis, data interpretation, or writing of the manuscript.

The authors vouch for the accuracy and completeness of the data and for the fidelity of the trial to the protocol, which is available online together with the full text of this article on the journal’s website. The trial was overseen by a data and safety monitoring board.

### Patients

Patients were eligible for enrolment if they were 18 years or older, capable of providing informed consent and had SARS-CoV-2 infection confirmed by nasopharyngeal swab polymerase chain reaction. Additionally, patients were required to be admitted to a ward and have at least one of the following signs compatible with hyperinflammation: 1) need for supplemental oxygen (inspired by the ASTCT consensus grade 2 for CRS, generally matching a saturation < 94%) [10] and/or 2) ferritin >2000ug/l or a doubling of serum ferritin in 20–48 hrs.

### Randomization and treatment

Patients were randomly assigned in a 1:1 ratio to receive standard of care with or without a single dose of tocilizumab (8 mg per kilogram of body weight administered intravenously, maximal 800 mg) via a web-based secure centralized system. A computer-generated assignment randomization list stratified by ICU eligibility and use of hydroxychloroquine and blocked with varying block sizes unknown to the investigators. A second dose of tocilizumab was permitted after 8 hours if hypoxia was not resolved (persisting at grade II or more according to CRS scale). Randomization was stratified according to the use of hydroxychloroquine and to (on admission) agreed restrictions for ICU eligibility (ICU eligible or ICU non-eligible).

### Concomitant treatment

The results of the Randomized Evaluation of Covid-19 Therapy (RECOVERY) trial [7] regarding the efficacy of dexamethasone became available during the trial. Therefore, the majority of patients (88%) received dexamethasone as a concomitant treatment. All other concomitants were permitted, including remdesivir and hydroxychloroquine.

### Outcomes

The primary outcome was 30-day mortality after randomization, assessed as a time-to-event endpoint. Patients still alive 30 days after randomization were censored at that point. Patients that withdrew informed consent were censored at the day of consent withdrawal. Secondary objectives included comparing the duration of hospital stay until 30 days after randomization, ICU admittance, duration of ICU stay until 30 days after randomization, and the duration of mechanical ventilation until 30 days after randomization. As a post-hoc analysis, we compared the composite endpoint time to mechanical ventilation and death by day 30 between the two arms, as this composite endpoint has been used in other tocilizumab trials in patients with Covid-19, such as the EMPACTA trial [11], in which the efficacy of tocilizumab was investigated in a population of COVID-19 patients with similar inclusion criteria as for our study.

### Statistical analysis

Based on early reports, we assumed a 30-day mortality of 20% in the control arm. To detect a decrease of 30-day mortality from 20% to 10% (hazard ratio (HR) = 0.472) with 80% power and a 2-sided significance level of α = 0.10, a total of 49 deaths were anticipated and 322 patients would need to be randomized 1:1. To accommodate a 10% margin for loss to follow up, a total of 354 patients were required. The 0.10 significance level was chosen because of the phase II design, in which a p-value below 0.10 would indicate that further investigation for efficacy would be warranted.

Unless explicitly stated otherwise, analyses were according to the intention-to-treat (ITT) principle, i.e., all randomized patients were analysed according to the treatment arm they were assigned to.

The primary analysis–the difference in 30-day mortality between the two arms–entailed Cox regression analysis with adjustment for only the stratification factor ICU-eligibility, because only 5 patients had received hydroxychloroquine. Hazard ratios (HR) and 95% confidence intervals (CI) were estimated, but because α = 0.10 was used the 90% CI was also determined for the primary endpoint. Reciprocal Kaplan-Meier curves (starting at 0% on day 0) were generated to illustrate mortality to day 30. The impact of tocilizumab treatment within subgroups according to age, ICU eligibility, CRP and ferritin was illustrated by forest plot. Discrete outcomes were compared between the two arms using the appropriate chi-squared test or Fisher exact test. The Mann-Whitney U test was used to compare continuous outcomes between the two arms. The number of days spent in hospital and at the ICU until 30 days after randomization were calculated from the day of admission (instead of day of randomization) until discharge, death or until 30 days after randomization, whichever came first. As a post-hoc analysis, patients who died before 30 days after randomization were considered in the analysis as being still admitted at 30 days after randomization, as suggested by Harhay et al. [12].The composite endpoint mechanical ventilation or death by day 30 was analysed similar to 30-day mortality. Except for the primary endpoint analysis, 95% CI’s were calculated. All analyses have been performed in Stata (StataCorp. 2019. Stata: Release 16. Statistical Software. College Station, TX: StataCorp LLC).

## Results

### Enrolment and randomization

**Fig 1:**
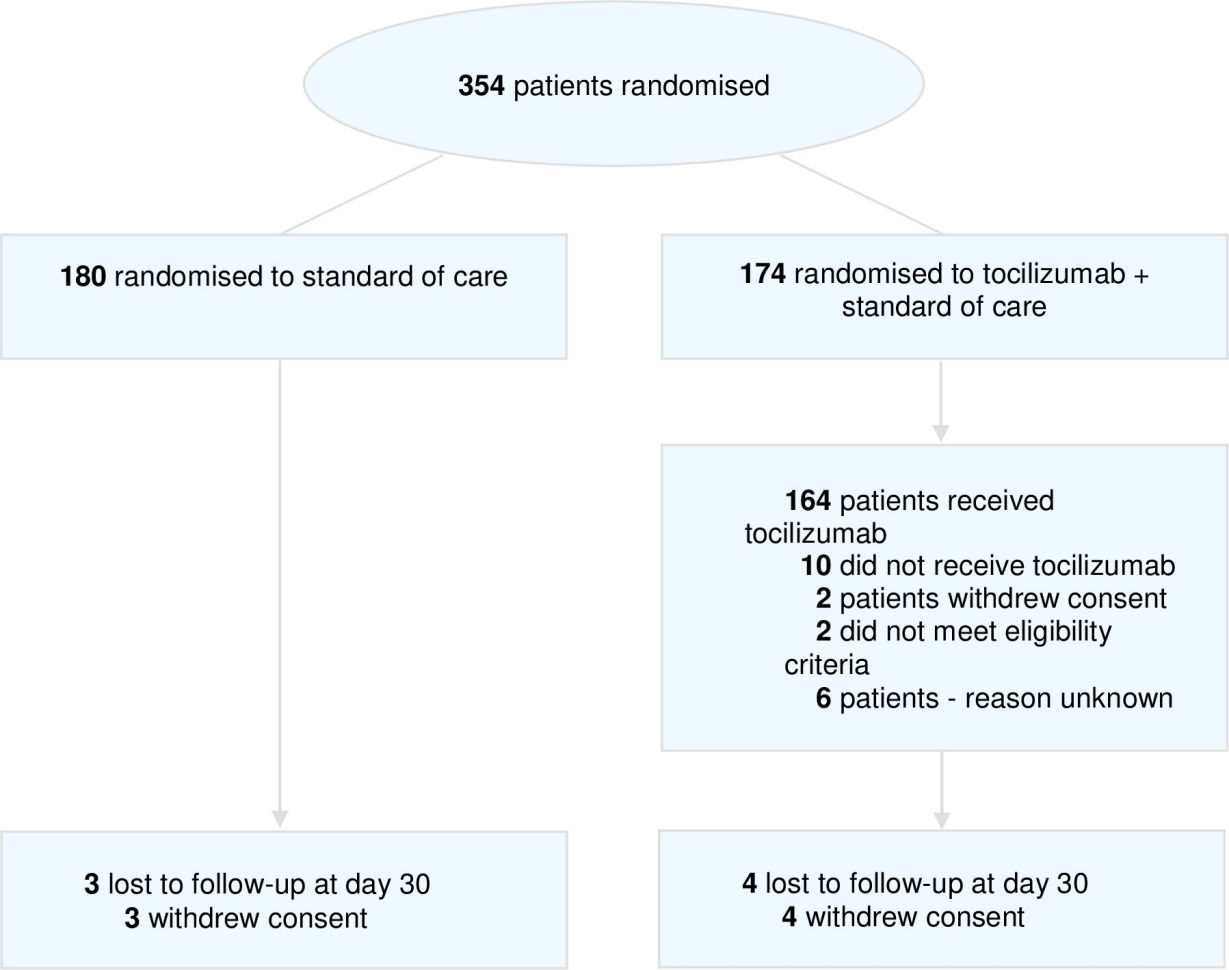
Study flow chart.

The first patient with Covid-19 in The Netherlands was diagnosed on February 27, 2020. The first patient in this trial was included on April 6, 2020, and the final patient was included on January 12, 2021. A total of 354 patients were randomized and all were included in the intention-to-treat population (Fig 1, 180 patients were randomized to standard of care (SOC) and 174 patients were randomized to SOC + tocilizumab (SOC+T). The majority of patients met the supplemental oxygen (342/354, 96.6%) inclusion criterion. A smaller proportion of the patients were eligible based on a high ferritin level (64/354, 18.1%). Twelve patients were eligible solely based on ferritin level, while no patients were included based solely on the ferritin doubling criterion. The intention to treat population included all 354 randomized patients. In the SOC+T arm, 164 of 174 patients (94.2%) received tocilizumab on day one, while 10 patients did not receive tocilizumab for various reasons including withdrawal of consent (2 patients), not meeting the eligibility criteria (2 patients), and unknown reasons (6 patients). Due to withdrawal of consent, 3 patients in the SOC group and 4 patients in the SOC+T arm were lost to follow-up from this point forward.

### Patients

Baseline characteristics were broadly comparable between the two arms (see Table 1). Median age was 66 years in the SOC arm and 67 years in the SOC+T arm. In both arms 67% were male. The median body mass index (BMI) was 29 kg/m^2^ in the SOC arm and 27 kg/m^2^ in the SOC+T arm. The grade of hypoxia at hospital presentation was also comparable, as 72% of patients in the SOC arm presented with grade II hypoxia versus 74% in the SOC+T arm. Percentages for grade III hypoxia were 24% and 22%, respectively, and grade IV hypoxia was 1% in both arms. ICU eligibility was also similar in both arms (78% versus 80%). Furthermore, medians for the inflammatory parameters C-reactive protein (CRP) and ferritin were 85 mg/L (SOC arm) and 75 mg/L (SOC+T arm) and 1003 ug/l (SOC arm) and 948 ug/l (SOC+T arm), respectively. At 8 days for time from onset of symptoms to hospitalization and 1 day for time of hospitalisation to randomization, these parameters were also comparable in the two arms. In total, 313 (88%) patients, equally distributed over both arms, received dexamethasone at randomization, 65 patients (18%) received remdesivir and 5 patients hydroxychloroquine (1.4%). The number of patients with comorbidities in the SOC arm was 136 (76%), compared to 127 (73%) in the SOC+T arm. A second dose of tocilizumab was given to 121 patients (74%).

**Table 1:** Baseline characteristics.

Variable	SOC (n = 180)	SOC+T (n = 174)
Age, ME (IQR)	66 (56–75)	67 (60–74)
Sex, n (%)		
Male	121 (67%)	116 (67%)
Female	59 (33%)	56 (33%)
unknown	-	2 (1%)
BMI (kg/m^2^), n (%)		
< 30	81 (45%)	96 (55%)
30–40	58 (32%)	46 (26%)
> 40	10 (6%)	8 (5%)
unknown	31 (17%)	24 (11%)
Hypoxia, n (%)		
Grade II	129 (72%)	128 (74%)
Grade III	44 (24%)	38 (22%)
Grade IV	1 (1%)	2 (1%)
unknown	6 (3%)	6 (3%)
ICU eligibility, n (%)	141 (78%)	139 (80%)
Ferritin, n (%)		
>2000 μg/L and/or doubling within 20–48 hours	36 (20%)	28 (16%)
Ferritin level D1 (μg/L), ME (IQR)	1003 (530–1869)	948 (461–1640)
CRP level D1 (mg/L), ME (IQR)	85 (48–135)	75 (43–132)
Lymphocyte count D1 (10^9^/L), ME (IQR)	0.70 (0.50–1.00)	0.70 (0.50–0.91)
Serum creatinine D1 (μmol/L), ME (IQR)	81 (64–98)	72 (63–84)
eGFR (ml/min/1.73m^2^), n (%)		
> 59	139 (77%)	145 (83%)
30–59	25 (14%)	20 (11%)
< 30	8 (4%)	2 (1%)
unknown	8 (4%)	7 (4%)
Time to hospitalization, ME (IQR) ^a^	8 (6–10)	8 (5–10)
Time to randomization, ME (IQR) ^b^	1 (1–2)	1 (1–2)
Dexamethasone given, n (%)	162 (90%)	151 (87%)
Hydroxychloroquine given, n (%)	4 (2%)	1 (1%)
Remdesivir given, n (%)	29 (16%)	36 (21%)
Comorbidities, n (%) ^c^	136 (76%)	127 (73%)

^a^ days between first symptoms and hospital admission

^b^ days between hospital admission and study randomization

^c^ e.g. malignancy, autoimmune disease, transplant

Abbreviations: SOC = Standard of care, SOC+T = SOC with tocilizumab, BMI = body mass index, CRP = C-reactive protein, eGFR = estimated glomerular filtration rate, ICU = intensive care unit. ME = median, IQR = interquartile range

### Primary efficacy outcome

**Fig 2:**
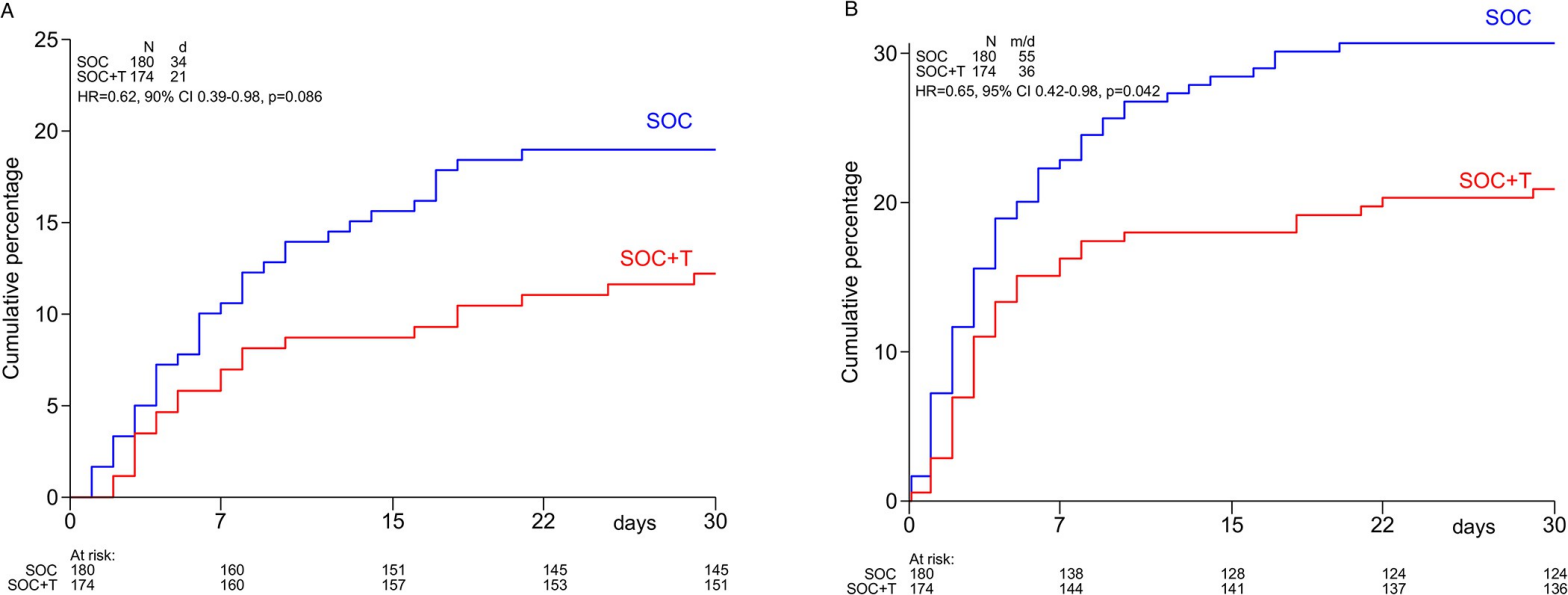
**A**. Mortality by treatment arm. SOC = standard of care, SOC+T = standard of care plus tocilizumab, N = number of patients, d = number of deaths, HR = hazard ratio, CI = confidence interval. **B**. Mechanical ventilation or death by treatment arm. SOC = standard of care, SOC+T = standard of care plus tocilizumab, N = number of patients, m/d = number of patients with mechanical ventilation or death, HR = hazard ratio, CI = confidence interval.

The cumulative percentage of patients who died by day 30, the primary endpoint of this study, was significantly lower in the SOC+T arm (12%, 95% CI 8%-18%) compared with the SOC arm (19%, 95% CI 14%-26%), resulting in a hazard ratio (HR) of 0.62 (90% CI 0.39–0.98; 95% CI 0.36–1.07; p = 0.086) (Fig 2A). Since the power calculation was based on a 2-sided significance level α of 10%, the primary objective of this study was met. Because 6 hospitals included less than 10 patients each, and one hospital included 185 patients, a post-hoc analysis was performed taking clustering by hospital into account, but this gave very similar results (HR = 0.62, 90% CI 0.41–0.94, p = 0.062).

### Secondary outcomes

The median duration of hospital stay was 9 days (IQR, 6–14) in the SOC arm, versus 9 days (IQR, 6–15) in the SOC+T arm (p = 0.80). Patients were included in the trial when they were hospitalized on a general ward, but in case of clinical worsening patients were transferred to the ICU if ICU eligible. The percentage of patients admitted to the ICU was the same in both arms (17%). Interestingly, the median duration of ICU stay was significantly shorter in patients treated with tocilizumab (9 days, IQR 5–14 days vs. 14 days, IQR 9–28, p = 0.014). In addition, fewer tocilizumab-treated patients required mechanical ventilation (10% in the SOC+T arm versus 15% the SOC arm), although this did not reach statistical significance (p = 0.18). Similarly, the median number of days on ventilation was significantly shorter in the SOC+T arm than in the SOC arm (10 days, IQR 7–12 vs. 15 days, IQR 9–26, p = 0.036). The post-hoc analyses, in which patients who died early were analysed as being still alive at day 30, gave similar results. Duration of hospital stay was still similar between the arms (median 11 days, IQR 7–31 in SOC vs. 10, IQR 6–19 in SOC+T, p = 0.31), while duration of ICU (median 9 days, IQR 6–14 in SOC+T vs. 17, IQR 12–30, p = 0.003) and mechanical ventilation (median 10 days, IQR 8–16 in SOC-T vs. 17, IQR 10–29, p = 0.036) were still significantly shorter in the patients treated with tocilizumab.

### Post-hoc analysis for efficacy

Following the publication of efficacy of tocilizumab in the EMPACTA trial [11], we performed a post-hoc analysis to approximate the endpoint of this trial, which was 28-day mortality or mechanical ventilation. The composite endpoint mechanical ventilation or death by day 30 in our study was significantly lower in the SOC+T arm (21%, 95% CI 16%-28%) compared with the SOC arm (31%, 95% CI 24%-38%), HR = 0.65 (95% CI 0.42–0.98; p = 0.042) (Fig 2B).

### Subgroup analyses

**Fig 3:**
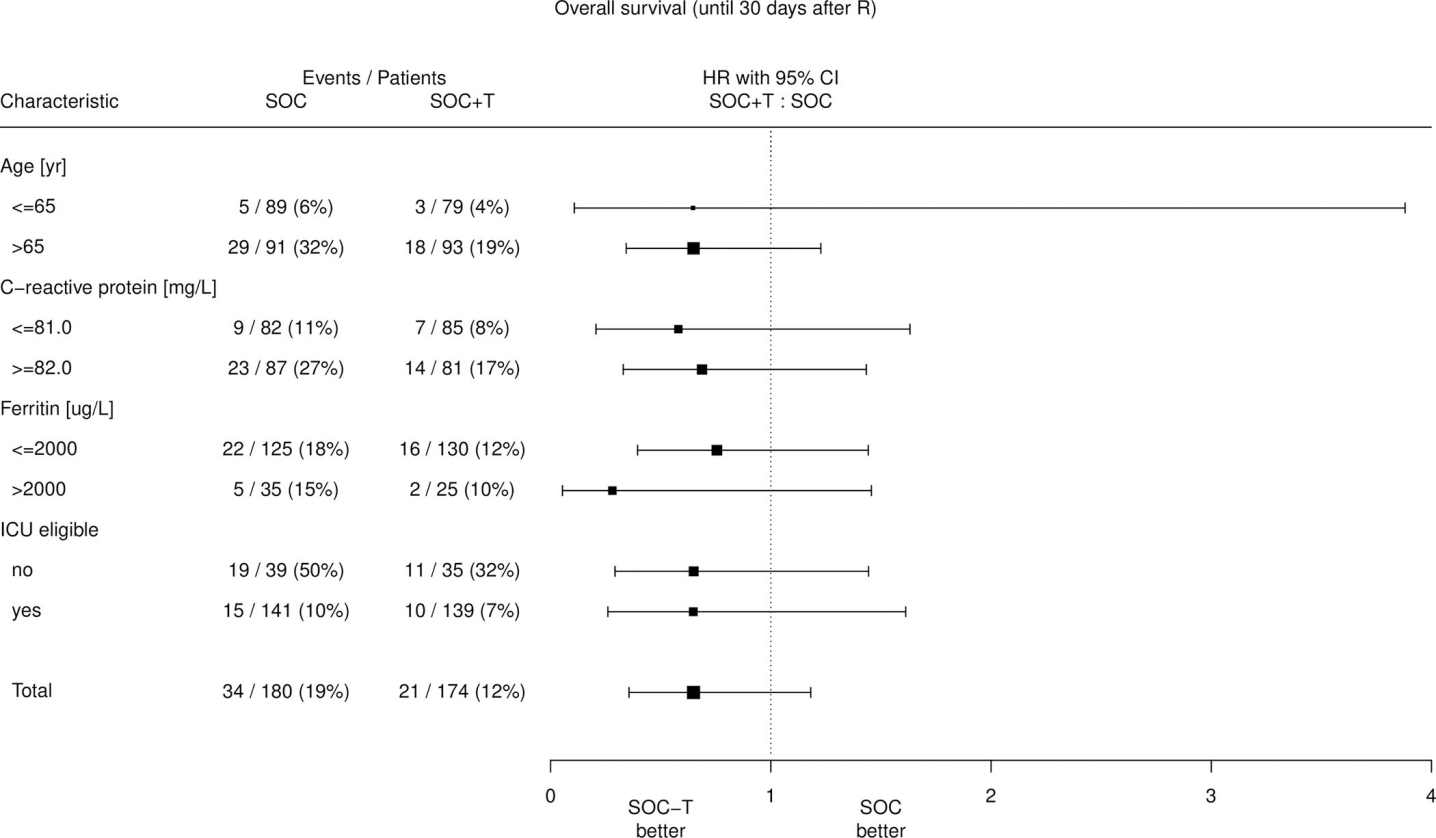
Forest plot. R = randomization, SOC = Standard of care, SOC+T = SOC with tocilizumab, ICU = intensive care unit, HR = hazard ratio, CI = confidence interval.

Subgroup analyses demonstrated benefit at all ages, although patients over the age of 79 with the highest mortality risk showed the most pronounced benefit. Compared to standard of care, tocilizumab showed benefit in both ICU eligible and non-eligible patients, as well as in patients with both high and low baseline CRP and ferritin (Fig 3).

### Safety

Adverse events of grade 4 or more were reported in 53 patients in the SOC arm and in 45 patients in the SOC+T arm (Tables 2 and 3). Through day 30, 7 cases of sepsis were noted, 5 in the SOC arm and 2 in the SOC+T arm. A total of 5 vascular events were observed (1 STEMI and 1 ischemic CVA in the SOC arm and 3 STEMIs in the SOC+T arm).

**Table 2:** Secondary outcomes at day 30.

Variable	SOC (n = 180)	SOC+T (n = 174)	*P* value
Hospitalization duration, ME (IQR)	9 (6–14)	9 (6–15)	0.80
ICU admission, n (%)	31 (17%)	29 (17%)	0.89
ICU admission duration, ME (IQR)	14 (9–28)	9 (5–14)	0.014
Mechanical ventilation, n (%)	27 (15%)	18 (10%)	0.18
Duration, ME (IQR)	15 (9–26)	10 (7–12)	0.036
AE ≥ grade 4, n (%)	53 (29%)	45 (26%)	0.45

Abbreviations: SOC = Standard of care, SOC+T = SOC with tocilizumab, IQR = interquartile range, ME = median, ICU = intensive care unit, AE = adverse event

**Table 3:** Adverse events.

Adverse event term	SOC (n = 180)	SOC+T (n = 174)
Thromboembolic event	4 (2%)	8 (5%)
STEMI	1	2
CVA	1	0
Pulmonary embolism	2	6
Bacteraemia	4 (2%)	3 (2%)
Sepsis	5 (3%)	2 (1%)

Abbreviations: SOC = Standard of care, SOC+T = SOC with tocilizumab, STEMI = acute ST-elevation myocardial infarction, CVA = cerebrovascular accident

## Discussion

Pre-emptive administration of tocilizumab in addition to the standard of care, including 88% use of dexamethasone, in hospitalized patients on supplemental oxygen resulted in a reduced risk of death and a reduced time spent in the ICU. This randomized phase II trial, including COVID-19 hospital patients on supplemental oxygen and/or with signs of hyperinflammation, met its primary endpoint. A post-hoc analysis supports clinical efficacy for reduction of mortality or mechanical ventilation at 30 days.

Eligibility criteria were inspired by ASTCT grading consensus criteria for cytokine release syndrome (CRS) [10]. CRS can be fatal if not recognized and treated promptly, and the ASTCT grading system is a simplified method developed to grade immune effector cell-associated CRS [10]. The need for oxygen supplementation corresponds to grade 2 or 3 CRS, depending on the level of hypoxia requiring oxygen via a low-flow nasal cannula (≤6 L/minute) (grade 2) or high-flow nasal cannula (>6 L/minute), facemask, non-rebreather mask or venturi mask (grade 3). In general, tocilizumab is advised for grade 2 immune effector cell CRS to prevent worsening of symptoms due to cytokine storm. In cases where patients do not improve following tocilizumab administration or immune effector cell CRS worsens to grade 3, dexamethasone is added to the treatment regime [13]. Our study was based on the treatment strategy devised for immune effector cell CRS and our data further support the notion that correct timing of tocilizumab administration is important. The inclusion criteria of our study captured the appropriate intervention moment, i.e. the interval between viral control and the exaggerated immune response that is primarily responsible for patient morbidity.

The first treatment breakthrough in COVID-19 was reported by results from the RECOVERY platform trial, which showed that a 10-day course of 6 mg of dexamethasone reduces mortality [7]. In this large, practice-changing trial that included 2104 patients, the mortality rate ratio was significantly reduced both in patients receiving invasive mechanical ventilation (rate ratio 0.64, 95% CI 0.51 to 0.81) and in patients receiving oxygen without invasive mechanical ventilation (rate ratio 0.82, 95% CI 0.72 to 0.94). Dexamethasone had no effect on patients who did not require respiratory support at randomization [7].

Several trials involving IL-6 antagonists tocilizumab or sarilumab for covid-19 have reported recently, with varying results (reviewed in [14]). Earlier studies by Salvarani [15], Hermine [16] and Stone [17] did not find a benefit for tocilizumab. These studies included patient populations with relatively low mortality in the control arms at day 28–30 (1.5%, Salvarani et al. [15]; 2.4%, Stone et al. [17]; 11.9%, Hermine et al. [16]) and reported limited use of steroids such as dexamethasone. The larger COVACTA study, including 452 patients, also failed to show a benefit of tocilizumab treatment despite a mortality rate of 19.4% [18]. A distinctive aspect of our study was that around 74% of patients received a second dose of tocilizumab, compared to only a quarter of patients in the COVACTA study. However, probably more importantly, only 19.4% (57/294) of patients in the COVACTA trial received both corticosteroids and tocilizumab versus 28.5% (41/144) receiving placebo and corticosteroids (chi-square test, p = 0.032). As discussed in an editorial [19], this approach differs from other studies that recently demonstrated a survival benefit of tocilizumab in both ICU patients (REMAP-CAP [20]) and non-ICU patients (EMPACTA trial [11]). In the EMPACTA trial, which included a population very similar to ours, the use of steroids was 80.3% in the tocilizumab arm and 87.5% in the control arm. Furthermore, a subanalysis performed by Hermine et al. found the largest reduction in mortality in tocilizumab-treated patients concomitantly treated with steroids [16]. In a large meta-analysis of trials, including prepublished data from ours, mortality reduction with tocilizumab was only significant in patient concomitantly treated with corticosteroids [14]. As the majority of our patients were included during the 2^nd^ COVID-19 wave, 87% of patients in the SOC+T arm in our study were treated with a combination of tocilizumab and dexamethasone. Combined IL-6 blockade, together with glucocorticoids, may be additive or even synergistic. For sarilumab, effects on outcome were generally less pronounced and the impact of corticosteroids less clear [14]. Importantly, based on the distribution of infectious complications and other SAEs, our data and other studies [14] indicate that the use of tocilizumab in COVID-19 infected patients is safe.

Further evidence for effectiveness of tocilizumab when added to corticosteroid treatment to reduce mortality from COVID-19 infection was brought by the RECOVERY platform trial, which included a second randomization where tocilizumab was compared to standard care [21]. In this part of the RECOVERY trial, concomitant corticosteroid use was 82%. Importantly, many patients were in a more advanced stage of COVID-19 disease as patients were included only after failure of standard treatment. Indeed, 54% was non-invasively or invasively ventilated at the time of inclusion. Therefore, an important difference with our trial is that tocilizumab was given earlier in the disease course. This may have contributed to the larger effect size on mortality found in our trial with a HR of 0.62 versus 0.85 in the RECOVERY trial. Consistently, the numeric HR in subgroup analyses of the RECOVERY trial was lowest in patients that did not receive ventilator support at time of inclusion, although this was not statistically significant. A further difference with the RECOVERY trial is that a CRP level >75 mg/l was required for inclusion. We included patients with any CRP level and found a similar effect size for those with a CRP below or above the median.

A limitation of our study is the phase II design with 2-sided significance level α of 10%. However, this study met its primary objective, and therefore signifying that further investigation of tocilizumab is warranted by its phase II results. Interpretation for clinical efficacy depends on integration with observations made by others. The best comparable study investigating tocilizumab in non-ICU patients receiving supplemental oxygen without mechanical ventilation and receiving steroids as part of standard of care is the EMPACTA trial [11]. This study showed a reduction in mortality or mechanical ventilation at day 28, which is fully in line with our post-hoc analysis for this endpoint at 30 days. Further support comes from the aforementioned meta-analysis of 27 randomized trials describing the effect of IL-6 antagonists in 10930 hospitalized COVID patients, including the data described in this manuscript [14]. Here, the summary odds ratios for the association of IL-6 antagonist treatment with 28-day all-cause mortality were 0.78 with concomitant administration of corticosteroids vs 1.09 without administration of corticosteroids.

Taken together, tocilizumab in combination with dexamethasone should be considered the new standard of care for all hospitalized COVID-19 patients requiring supplemental oxygen. This treatment strategy will help limit use of ICU capacity and, most importantly, reduce the risk of COVID-19-related morbidity and death. Our trial supports further investigation towards the administration of tocilizumab early in the course of COVID-19 disease in patients requiring any level of oxygen supplementation and irrespective of CRP level.

## Supporting information

S1 Checklist Consort checklist. (PDF)
